# Changes in mandibular radiomorphometric indices in osteoporosis patients treated with denosumab: a retrospective case-control study

**DOI:** 10.1186/s12903-024-03870-1

**Published:** 2024-01-16

**Authors:** Katia Rupel, Chiara Dal Broi, Giulia Ottaviani, Laura Bellassai, Theodora Magdalena Bogdan Preda, Roberto Di Lenarda, Matteo Biasotto

**Affiliations:** Department of Medicine, Surgery and Health Sciences, University of Trieste, Strada di Fiume, n 447 - 34129, Trieste, Italy

**Keywords:** Bone Mineral Density, Dental panoramic radiography, Bone metabolism markers, Antiresorptive therapy, Denosumab

## Abstract

**Background:**

Radiomorphometric indices measured on Dental Panoramic Radiography (DPR) can reflect Bone Mineral Density (BMD). The aim of our study is to evaluate changes in DPR radiographic markers in patients undergoing antiresorptive therapy with denosumab and correlate them to BMD and serum bone turnover markers (BTM).

**Methods:**

We evaluated two radiomorphometric indices: Mandibular Cortical Width (MCW) and Panoramic Mandibular Index (PMI), in patients undergoing antiresorptive therapy with denosumab at T0 (before starting the therapy) and at T1 (after 12 months), comparing results with a control group of healthy patients who performed two DPRs at a one-year time distance. Correlation analysis was performed in the denosumab group, as well as ROC curves were obtained for both indices.

**Results:**

The study included 18 patients and 21 controls according to specific inclusion and exclusion criteria, matched by gender and age. Both MCW and PMI were significantly lower at T0 in the denosumab group, consistently with lower BMD. MCW showed significant correlation with femoral and lumbar DEXA and was significantly lower in patients with osteoporosis compared to osteopenia. Only PMI index increased significantly in the denosumab group from T0 to T1. After one year (T1), there weren’t any differences between patients and controls for both indices. No significant correlations were found with BTMs. Sensitivity and specificity for MCW and PMI were also calculated.

**Conclusions:**

Our results show how CMW shows sufficient sensitivity and specificity to be used as a radiographic marker to screen and intercept patients with osteoporosis. PMI seems to be able to reflect changes in response to antiresorptive therapy with denosumab. Further studies are needed to confirm our hypothesis.

## Background

Osteoporosis is a disease characterized by a reduction in bone mass and a deterioration of the microarchitecture of bone tissue, therefore enhancing bone fragility, with a consequent increase in the risk of fracture [1]. The gold standard procedure employed in the measurement of Bone Mineral Density (BMD), and therefore to perform diagnosis of osteoporosis or osteopenia, is Dual Energy X-Ray Absorptiometry (DEXA). The differences between the reference BMD values, defined by the World Health Organization (WHO), and the measured individual values are expressed as Standard Deviations (SD) scores and defined as T-scores. A T-score value between − 1.0 and − 2.5 SD is defined as osteopenia, and values equal to or lower than − 2.5 SD assesses a diagnosis of osteoporosis [2].

In recent years, it has been investigated whether radiographs performed for dental purposes, in particular dental panoramic radiography (DPR), could play a role in the diagnosis of pathologies involving changes in BMD. Various radiomorphometric indices have been taken into consideration such as the mandibular cortical width (MCW), panoramic mandibular index (PMI), mental index (MI), antegonial index (AI) and gonial index (GI) [3–7]. In fact, several studies have correlated the radiomorphometric indices identified in DPR with lower BMD and increased risk of osteoporosis [8–10]. A recent systematic review and meta-analysis assessed how reproducibility is rated as almost perfect and substantial for MI, MCW and PMI in both intra- and interexaminer agreement, but none of the indices described so far has the ideal sensitivity and specificity for identifying reduced BMD alone. Therefore, they can be a useful screening tool to be used in combination with clinical parameters [11].

Denosumab is a recently introduced antiresorptive drug, used both in metabolic and in neoplastic bone metabolism disorders, that inhibits receptor activator of NF-κB ligand (RANKL) [12]. Placebo-controlled clinical trials showed that therapy with denosumab in post-menopausal women suffering from osteoporosis increases BMD, decreases biochemical markers of bone remodeling, and reduces the risk of fractures [13, 14].

According to literature published so far, we hypothesize that changes in BMD caused by anti-resorptive therapy with denosumab could be detected by measuring mandibular radiomorphometric indices on DPRs after 12 months of treatment. Here we present the preliminary results of a study aimed at evaluating changes in two mandibular radiomorphometric indices (MCW and PMI) measured on DPRs in relation to the antiresorptive therapy compared to healthy subjects, and their correlation with clinical variables, serum bone turnover markers and BMD measured with DEXA before starting antiresorptive treatment and after 12 months. To our knowledge, this is the first study to describe changes over time in mandibular radiomorphometric indices with relation to antiresorptive therapy with denosumab.

## Methods

### Study design and population

The design of the study was retrospective case control, and it was conducted in accordance with the principles outlined in the Declaration of Helsinki. The protocol was reviewed and approved by the University of Trieste Ethics Committee (129/2023).

To be eligible for the study, subjects had to meet specific characteristics. Inclusion criteria were:

- Subjects who underwent yearly oral evaluations with the execution of DPRs at the Dental Clinic of the University of Trieste for at least 2 years using the same X-ray machine (Myray Hyperion X9, Cefla dental group, Imola (BO), Italy) at 7 mA, 12.7s and 74.0 kV voltage using automatic exposure control. Images were stored in JPEG format with a matrix of 2620 × 1501 pixels.
- Diagnosis of osteopenia (T score between − 1 and − 2.5) or osteoporosis (T score > − 2.5) measured with DEXA and bone metabolism markers (ALP, Vitamin D, Phosphorus, Calcium, PTH) about to start antiresorptive therapy with denosumab.
- Age 18 ≥ years.
- Patients who have given consent to the use of their clinical data, made anonymous, for the purpose of clinical and epidemiological research.

Exclusion criteria were:

- Subjects with systemic diseases that could interfere or modify bone metabolism (diabetes, chronic kidney disease).
- Subjects with current or previous therapy with antiresorptive, bone builder or corticosteroid drugs.
- Subjects with previous head/neck Radiotherapy.
- Smokers.

Subjects meeting all inclusion and exclusion criteria were assigned to the test group (DEN). Healthy subjects meeting all criteria except for diagnosis of osteopenia/osteoporosis and denosumab treatment were assigned to the control group (CTRL).

### Clinical variables and mandibular radiomorphometric indices

The diagnosis of bone metabolism disorders was carried out by a physician specialized in Internal Medicine/Orthopedics/Endocrinology following the most recent guidelines [15]. All patients included in the DEN group performed a DEXA and measured specific serum markers before starting therapy with denosumab and after 1 year. Serum markers included in the study were the following: Alkaline phosphatase (ALP), Parathyroid hormone (PTH), Vitamin D (Vit D), Calcium (Ca), Phosphorus (P). BMD was measured according to the World Health Organization standardization, and patients were assigned basing on their T-score as follows: osteopenia with a T-score between − 1 and − 2.5 SD, osteoporosis with a T-score of -2.5 SD and lower [2].

A preliminary dental evaluation was requested to perform all necessary dental treatments to prevent the onset of Medication-Related Osteonecrosis of the Jaws (MRONJ) [16, 17]. Therefore, DPRs were taken at T0 (before starting the antiresorptive therapy) and T1 (after one year) in the DEN group. In the CTRL group, two DPRs were taken for dental treatment purposes at a 1-year distance. The DPRs were analyzed by a single operator through Image J software (Rasband, W.S., ImageJ, U. S. National Institutes of Health, Bethesda, Maryland, USA, https://imagej.nih.gov/ij/, 1997–2018.). The MCW and PMI indices were calculated as follows:

- MCW was calculated as cortical width along a line passing through the center of the mental foramen and perpendicular to the tangent to the lower border of the mandible [6].
- PMI was calculated as the ratio of the mandibular cortical thickness measured on the line perpendicular to the bottom of the mandible, at the middle of mental foramen, to the distance between the superior margin of inferior mandibular cortex and bottom of the mandible [7].

**Fig. 1:**
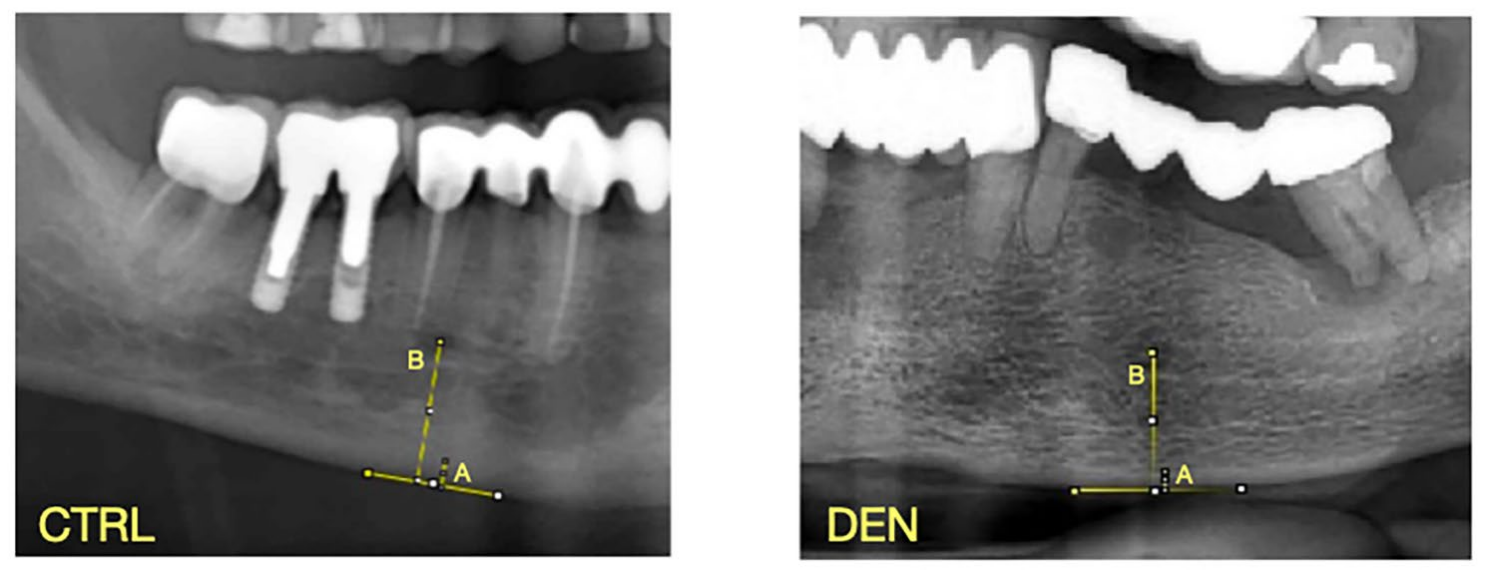
Mandibular radiomorphometric indices calculation examples for DEN and CTRL group. MCW = A; PMI = A/B.

The procedure is explained in Fig. 1.

### Statistical analysis

The statistical analysis was performed with the software Prism® (version 9.1.0, GraphPad Software, Inc., 7825 Fay Avenue, Suite 230, La Jolla, CA 92,037 USA). The normality of data distribution was evaluated employing the Kolmogorov-Smirnov test. The data were distributed non-normally; therefore, non-parametric statistical tests were used.

The Mann-Whitney U test was used to evaluate the differences between the 2 groups, Wilcoxon paired test to analyze changes in clinical parameters between T0 and T1 within each group. The Chi-square test was used to analyze the significance of the differences in categorical variables between groups. Spearman’s test was used to perform correlation analyses between continuous variables in the DEN group. ROC curve analysis was used to measure the diagnostic accuracy of PMI and MCW for diagnosis of osteoporosis or osteoporosis/osteopenia. The areas under ROC curves (A_z_) represent the probability that a randomly selected individual from the patient group has a test result indicating greater risk of osteoporosis than that for a randomly chosen individual from the control group. In addition, sensitivity and specificity parameters were obtained. A *p* value < 0.05 was used for the rejection of the null hypothesis.

## Results

### Demographic characteristics of subjects

The total number of subjects included in the study was 39. 18 patients were included in the DEN group, and 21 in the CTRL group. The mean age of the DEN and CTRL groups were 74 ± 10 years, and 66 ± 18 years, respectively. We didn’t find significant differences in mean age between groups (Mann- Whitney U test p = NS), or in gender distribution (Chi-squared test p = NS).

Among the DEN group, 61% patients were diagnosed with osteoporosis according to DEXA values, and 39% patients were diagnosed with osteopenia. Radiomorphometric and serum markers, as well as femoral and lumbar DEXA values at T0 (before starting the antiresorptive therapy) and T1 (after 1 year) are reported in Table 1.

**Table 1:** Radiomorphometric indices, DEXA values and serum markers in the DEN group at T0 and T1. Data expressed as mean ± standard deviation. ^a^ Wilcoxon signed-rank test

	T0	T1	*p*-value
**PMI**	0.26 ± 0.08	0.31 ± 0.10	*p* < 0.0001
**MCW**	10.13 ± 4.7	12.00 ± 8.88	NS
**DEXA femoral**	-2.19 ± 1.0	-2.26 ± 0.73	*p* = 0.0273
**DEXA lumbar**	-1.62 ± 2.04	-1.96 ± 0.83	NS
**Akaline Phosphatase**	70.23 ± 18.71	56.27 ± 19.96	NS
**Parathormone**	59.31 ± 35.87	41.68 ± 16.03	NS
**Phosphorus**	3.37 ± 0.54	3.37 ± 0.65	NS
**Calcium**	9.63 ± 0.34	9.62 ± 0.54	NS
**Vitamin D**	28.91 ± 15.72	35.64 ± 9.51	NS

### Changes in radiomorphometric indices in DEN versus CTRL subjects

**Fig. 2:**
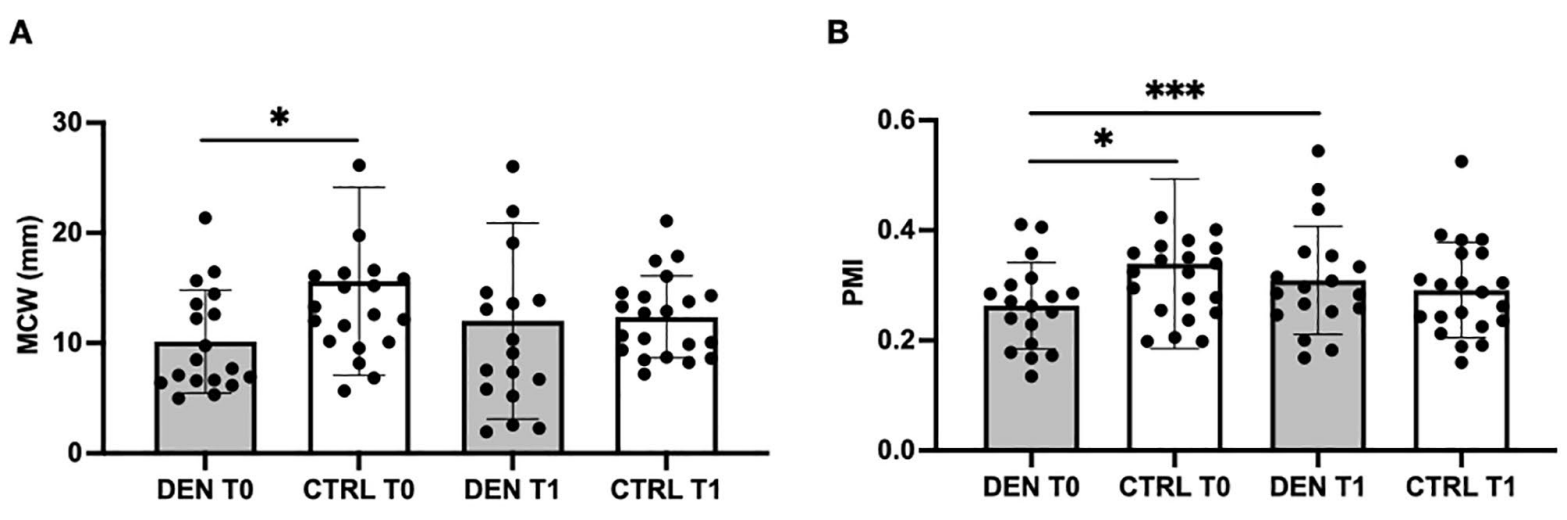
Changes in mandibular radiomorphometric indices after 1 year of therapy with denosumab, patients (DEN) versus controls (CTRL). **A**. MCW at T0 and T1 in DEN and CTRL groups. **B**. PMI at T0 and T1 in DEN and CTRL groups. DEN and CTRL at T0 versus T1: Wilcoxon signed-rank test. DEN versus CTRL at T0 and T1: Mann-Whitney U test. * *p* < 0.05 *** *p* < 0.0001

As described in the Methods section, we evaluated changes in the radiographic markers MCW and PMI at T0 and T1 evaluating the differences between groups (DEN and CTRL), and within each group for the two time points. We found a significant difference in both PMI and MCW values between the DEN and CTRL group at T0 (Mann-Whitney test U test *p* = 0.0358 for MCW and *p* = 0.0104 for PMI), while there were no significant differences at T1 (Mann-Whitney test U test p = NS). We didn’t find significant differences between T0 and T1 in neither group for MCW (Wilcoxon signed-rank test p = NS). Notably, we observed a significant variation from T0 to T1 in both groups for PMI. Specifically, in the DEN group there was a significant increase in the PMI value (Wilcoxon signed-rank test *p* < 0.0001), while in the CTRL group we found a significant, albeit slight, reduction (Wilcoxon signed-rank test *p* = 0.0013). Results are represented in Fig. 2A-B.

### Changes in serum bone metabolism markers and DEXA values in the DEN group

We did not find significant changes between T0 and T1 for any of the considered serum bone metabolism markers (Wilcoxon signed-rank test p = NS), as reported in Table 1.

Considering DEXA measured at femoral and lumbar level, we found a significant decrease in femoral DEXA T-score between T0 and T1 (Wilcoxon signed-rank test *p* = 0.0273), while there was no significant change in lumbar DEXA T-score (Wilcoxon signed-rank test p = NS), as represented in Table 1.

### Differences in radiomorphometric indices according to diagnosis of osteoporosis/osteopenia

Subsequently, we divided the DEN group into two subgroups according to the femoral T- score value at T0 in patients with a T-score between − 1 and − 2.5 (osteopenia) and less than − 2.5 (osteoporosis) and assessed possible differences in MCW and PMI.

**Fig. 3:**
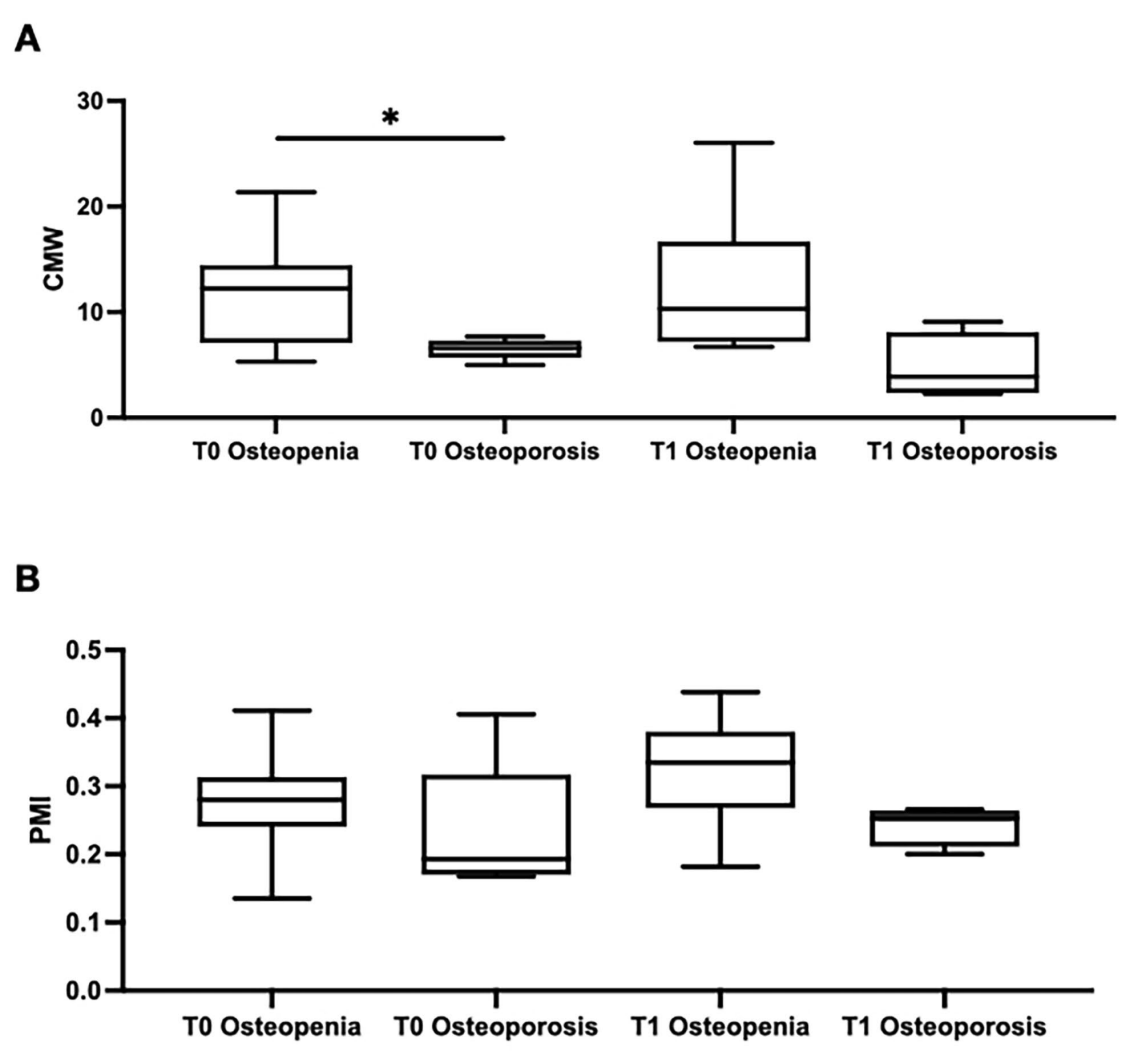
Changes in mandibular radiomorphometric indices in DEN group comparing patients with osteopenia (T-score between − 1 and − 2.5) and osteoporosis (T-score lower than − 2.5). **A**. MCW at T0 and T1 in Osteopenia and Osteoporosis groups. **B**. PMI at T0 and T1 in Osteopenia and Osteoporosis groups. Osteopenia and Osteoporosis at T0 versus T1: Wilcoxon signed-rank test. Osteopenia versus Osteoporosis at T0 versus T1: Mann-Whitney U test. * *p* < 0.05

With regards to PMI, we did not detect any significant differences between patients with osteopenia and osteoporosis either at T0 or at T1 (Mann-Whitney U test p = NS), even if we observed how lower T-score values (i.e., osteoporosis) also have lower PMI values. When considering the MCW parameter, we found a significant difference between osteopenia and osteoporosis (Mann-Whitney U test *p* = 0.0192). In fact, subjects with lower T-score values also have significantly lower MCW values. Results are represented in Fig. 3A-B.

### ROC analysis, sensitivity and specificity of MCW and PMI

**Fig. 4:**
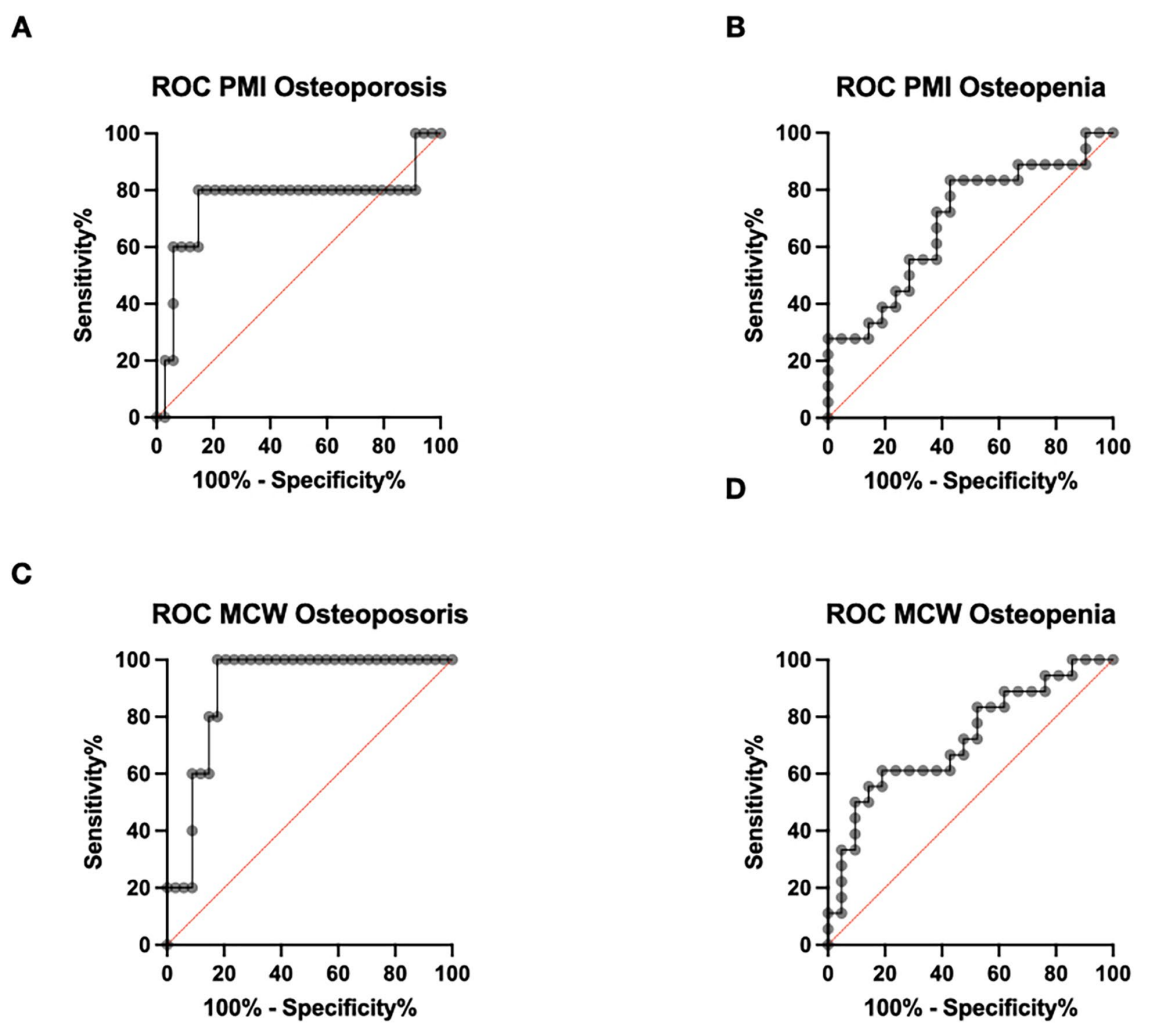
ROC curves representing the diagnostic performances of PMI and MCW in the diagnosis of Osteoporosis and Osteopenia

Four ROC curves were constructed to determine the diagnostic validity of MCW and PMI in the diagnosis of osteoporosis or osteopenia, respectively, as represented in Table 2; Fig. 4A-D. The highest A_z_ values were seen in the ROC curves constructed for the diagnosis of Osteoporosis with both indices. In the case of MCW, a cut-off threshold value ≤ 8 mm had the highest performance (100% sensitivity, 79% specificity), while in the case of PMI the best performance was given by a cut-off threshold value ≤ 0.23 (80% sensitivity, 85% specificity). In the case of Osteopenia, both indices had lower performances.

**Table 2:** Diagnostic validity of MCW and PMI at different cut-off values in the diagnosis of Osteoporosis or Osteopenia, showing the area under curve (A_z_) values for ROC curves, sensitivity and specificity (with 95% confidence intervals)

	Osteoporosis		Osteopenia	
**A**_**z**_ **(95% CI)**	0.7588 (0.4551-1.000)		0.6878 (0.5178–0.8579)	
	Sensitivity (95% CI)	Specificity (95% CI)	Sensitivity (95% CI)	Specificity (95% CI)
**PMI ≤ 0.23**	80.00 (37.55–98.97)	85.29 (69.87–93.55)	27.28 (12.50-50.87)	87.71 (65.36–95.02)
**PMI ≤ 0.3**	80.00 (37.55–98.97)	44.12 (28.88–60.55)	72.22 (49.13–87.50)	57.14 (36.66–75.53)
**A**_**z**_ **(95% CI)**	0.9000 (0.8008–0.9996)		0.7222 (0.5594–0.8850)	
	Sensitivity (95% CI)	Specificity (95% CI)	Sensitivity (95% CI)	Specificity (95% CI)
**MCW ≤ 8**	100.0 (56.55–100.0)	79.41 (63.20-89.65)	50.00 (29.03–70.97)	90.48 (71.09-98-31)
**MCW ≤ 10**	100.0 (56.55–100.0)	70.59 (53.83–83.17)	61.11 (38.62–79.69)	76.19 (54.91–89.37)

### Correlation analysis of radiomorphometric indices, DEXA and serum markers

We employed the Spearman’s correlation test to evaluate possible associations among continuous variables at T0 and T1. We included serum variables (PTH, ALP, Vit D, Calcium, Phosphorus), radiomorphometric indices (PMI, MCW) and femoral and lumbar DEXA values.

Significant correlations were found at T0 between: MCW and lumbar DEXA (*p* = 0.019; *r* = 0.57), MCW and femoral DEXA (*p* = 0.001; *r* = 0.75), Lumbar DEXA and femoral DEXA (*p* = 0.016; *r* = 0.60), Lumbar DEXA and Ca (*p* = 0.016; *r* = -0.69), Ca and PTH (*p* = 0.049; *r* = -0.58). At T1, statistically significant correlations were found between: PMI and MCW (*p* = 0.007; *r* = 0.64), Ca and P (*p* = 0.042; *r* = 0.55).

**Fig. 5:**
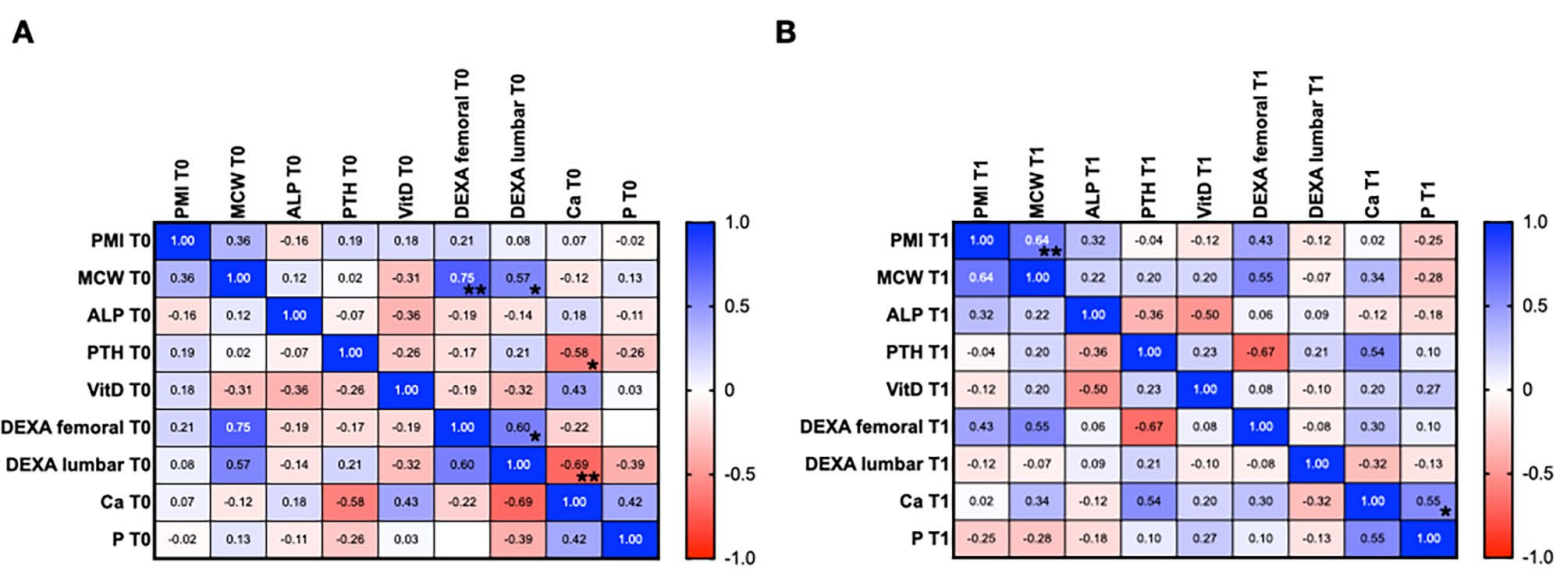
Correlation analysis of radiomorphometric indices with DEXA and serum markers. Data are represented as r-values at T0 (**A**.) and T1 (**B**.). We employed the Spearman’s correlation test to evaluate possible association among continuous including serum markers (*PTH* Parathormone, *ALP* Alkaline Phosphatase, *Vit D* Vitamin D, *Ca* Calcium, *P* Phosphorus), radiomorphometric indices (PMI, MCW) and femoral and lumbar DEXA values. A positive r-value indicates a positive correlation, where the values of the two variables tend to increase in parallel. A negative r-value indicates a negative correlation. The closer the color of the cell is to blue or deep red, the stronger the correlation, whether it is positive or negative. Significant correlations with * *p* < 0.05, ** *p* < 0.001

All results are represented in the correlation matrixes included in Fig. 5. In the T1 matrix a strong, although not significant, positive correlation between MCW and femoral DEXA (*r* = 0.55), can be observed.

## Discussion

DPRs are routinely performed instrumental evaluations in clinical dental practice, and mandibular radiomorphometric indices are becoming increasingly popular tools for screening or preliminary diagnosis of osteopenia and osteoporosis, especially due to their easy and reproducible measurement. In this context, DPRs are always performed in patients suffering from both neoplastic and metabolic bone disorders candidate to antiresorptive therapies with bisphosphonates or denosumab, in order to schedule preliminary dental treatments with the aim of reducing the risk of developing MRONJ [16–18], and they are often repeated at follow-ups.

Up to date, DEXA remains the standard method used to diagnose and monitor the efficacy of the therapies in metabolic bone disorders, and it is usually repeated every 2 years [19]. It is often associated to serum bone turnover markers, which are biochemical indicators that measure the level of bone formation and/or resorption. Despite being easily measurable at serum level, their diagnostic and prognostic significance are still a matter of debate today and there is a lack of consensus for their clinical use with the purpose of monitoring the efficacy of antiresorptive or bone builder therapies [20].

In the present study we measured two mandibular radiomorphometric indices in a cohort of patients with osteopenia/osteoporosis about to begin antiresorptive therapy with denosumab comparing them with healthy subjects, evaluated changes after 1 year, also considering the values of femoral and lumbar DEXA and serum bone metabolism markers to detect possible correlation trends among these variables. Among the indices described so far, we chose to use PMI and MCW, considered among the most accurate and reproducible radiomorphometric indices in DPRs based on published literature [3, 11].

Before starting the antiresorptive therapy, we found that both PMI and MCW values were significantly lower in the group of patients affected by osteopenia/osteoporosis compared to healthy controls, matched by age and gender. Thus, both radiomorphometric indices were able to detect a lower BMD. In particular, correlation analysis confirmed that MCW resulted in having a significant positive correlation to both DEXA lumbar and femoral T-scores. When patients in the DEN group were subdivided by BMD, both PMI and MCW showed even lower values in patients with a T-score lower than − 2.5, i.e., with a diagnosis of osteoporosis, when compared to patients with osteopenia (with a T-score between − 1 and − 2.5). These results are consistent with other studies previously published [21–27]. We determined the cut-off values with the best diagnostic performances in determining a diagnosis of osteoporosis, and obtained a value of ≤ 0.23 for PMI, which is lower than previously described in literature, where most studies reported cut-off values of ≤ 0.3 to 0.4 [10, 28]. Regarding MCW, we measured higher values than previously published research [29, 30] and therefore our threshold values were accordingly more elevated, with the best performance obtained for ≤ 8 mm in the identification of osteoporosis patients. In general, the levels of sensitivity and specificity of the method depend on the selected cut-off threshold value. From our results, MCW was overall the most reliable index in the diagnosis of reduced BMD, consistently with previous studies [29, 31].

To our knowledge, this is the first study that evaluated changes in radiomorphometric indices on DPRs in patients on antiresorptive therapy with denosumab. Interestingly, the PMI significantly increases over time in the group of osteoporosis/osteopenia patients one year after the beginning of the antiresorptive therapy, and slightly (but significantly) decreases in the control group. The latter finding is consistent with the study by Ledgerton et al. [4], describing how radiomorphometric indeces gradually decrease with age up to the sixth decade. The fact that PMI increases over time during denosumab treatment is likely related to the decrease in osteoclast activity associated with antiresorptive therapy, which leads to an increase in new bone deposition by osteoblasts, observed in the thickness of the mandibular cortex. Recent studies have shown that treatment with denosumab progressively increases BMD, especially in the first five years of treatment, then tends to a plateau [32–34]. MCW showed the same trend over time, but the changes were not significant. When comparing PMI and MCW in patients versus controls at T1, we didn’t find significant differences, showing possibly that BMD in the patients’ group increased to the point that it became superimposable to the bone mineral density found in healthy controls, thus potentially confirming the efficacy of antiresorptive therapy.

When considering serum markers of bone metabolism, we didn’t find any significant changes over time in the patients’ group. In addition, no correlations were found between any of the serum markers considered (ALP, PTH, Vitamin D, Ca and P) and radiomorphometric indices. The role of serum markers for monitoring bone metabolism and antiresorptive therapies is still unclear, as reported in several recommendation and scientific works [35, 36].

DPRs are widely used as source of information about oral health as they visualize all teeth and surrounding structures in one image, but their analysis if performed manually may be time-consuming. For this purpose, several studies have investigated the use of artificial intelligence (AI) algorithms on DPRs as an aid to diagnosis and/or treatment planning. A recent overview of systematic reviews described how among the studies that analyzed the use of AI for osteoporosis detection, especially convolutional neural network turned out to be a reliable tool for automated osteoporosis screening [37, 38]. In the context of this research, we didn’t employ AI in analyzing panoramic images, but such results certainly encourage the execution of larger studies where it would be useful to use AI to automate and speed up the analysis.

One of the most important adverse effects of antiresorptive therapies is MRONJ and to date, in fact, there is still no molecular or instrumental screening method to identify subjects at increased risk [17, 18]. Future developments of this study also include the evaluation of possible correlations between radiomorphometric indices and MRONJ development, in order to contribute to the identification of patients at increased risk.

## Conclusions

With the limitation of the sample size, this research fits into the context of the already numerous studies that confirm the possibility of using radiomorphometric indices, easily detectable in DPRs, and a condition of decreased BMD, in order to help the clinician in intercepting the patient with skeletal related diseases. In fact, in many patients the condition of osteoporosis remains undiagnosed until a pathological fracture occurs, due to lack of controls and screening or in countries where, because of the high cost, it is difficult to carry out a DEXA exam [39]. For this purpose, MCW seems to have the highest accuracy.

In addition, we report how the same indices could be considered as useful tools for monitoring of the response to antiresorptive therapy. Our results point out how especially PMI was able to detect significant changes 1 year after the beginning of the therapy. In case of ascertained diagnosis of osteoporosis or osteopenia, the measurement of the radiomorphometric indices could be included among the clinical and instrumental evaluations to be performed on the patient before starting the antiresorptive therapy, in order to contribute to the monitoring of the therapy itself. Therefore, the study represents an important advance compared to the state of the art since it is the first to evaluate any changes in radiomorphometric indices on DPRs in response to antiresorptive therapy.

To our knowledge, this is the first study describing such findings. The results of the study may encourage the organization of studies with a greater number of subjects, possibly using automatized AI approaches. Once standardized, this method could be easily translated into the application in daily clinical activity.

## Data Availability

No datasets were generated or analysed during the current study.
